# Dyslexic brain activation abnormalities in deep and shallow orthographies: A meta‐analysis of 28 functional neuroimaging studies

**DOI:** 10.1002/hbm.23202

**Published:** 2016-04-07

**Authors:** Anna Martin, Martin Kronbichler, Fabio Richlan

**Affiliations:** ^1^ Centre for Cognitive Neuroscience, University of Salzburg Hellbrunnerstr. 34 Salzburg 5020 Austria; ^2^ Department of Psychology University of Salzburg Hellbrunnerstr. 34 Salzburg 5020 Austria; ^3^ Neuroscience Institute, Christian Doppler Clinic, Paracelsus Medical University Ignaz‐Harrer‐Str. 79 Salzburg 5020 Austria

**Keywords:** dyslexia, fMRI, language, orthographic depth, PET, reading

## Abstract

We used coordinate‐based meta‐analysis to objectively quantify commonalities and differences of dyslexic functional brain abnormalities between alphabetic languages differing in orthographic depth. Specifically, we compared foci of under‐ and overactivation in dyslexic readers relative to nonimpaired readers reported in 14 studies in deep orthographies (DO: English) and in 14 studies in shallow orthographies (SO: Dutch, German, Italian, Swedish). The separate meta‐analyses of the two sets of studies showed universal reading‐related dyslexic underactivation in the left occipitotemporal cortex (including the visual word form area (VWFA)). The direct statistical comparison revealed higher convergence of underactivation for DO compared with SO in bilateral inferior parietal regions, but this abnormality disappeared when foci resulting from stronger dyslexic task‐negative activation (i.e., deactivation relative to baseline) were excluded. Higher convergence of underactivation for DO compared with SO was further identified in the left inferior frontal gyrus (IFG) pars triangularis, left precuneus, and right superior temporal gyrus, together with higher convergence of overactivation in the left anterior insula. Higher convergence of underactivation for SO compared with DO was found in the left fusiform gyrus, left temporoparietal cortex, left IFG pars orbitalis, and left frontal operculum, together with higher convergence of overactivation in the left precentral gyrus. Taken together, the findings support the notion of a biological unity of dyslexia, with additional orthography‐specific abnormalities and presumably different compensatory mechanisms. The results are discussed in relation to current functional neuroanatomical models of developmental dyslexia. *Hum Brain Mapp 37:2676–2699, 2016*. © **2016 The Authors Human Brain Mapping Published by Wiley Periodicals, Inc**.

## INTRODUCTION

There has been considerable effort in understanding the neurobiological basis of developmental dyslexia. During the last two decades, a substantial number of studies using neurocognitive methods such as functional magnetic resonance imaging (fMRI) and positron emission tomography (PET) were conducted to reveal the neural systems linked to difficulties in learning to read [e.g., Blau et al., 2009; Eden et al., 1996; van Ermingen‐Marbach et al., 2013; Hoeft et al., 2007; Hu et al., 2010; van der Mark et al., 2009; Richlan et al., 2010; Schurz et al., 2014c; Shaywitz et al., 2002; Temple et al., 2003; Wimmer et al., 2010]. Findings were summarized in both narrative reviews [e.g., Démonet et al., 2004; Heim and Keil, 2004; McCandliss and Noble, 2003; Pugh et al., 2000; Richlan, 2012; Sandak et al., 2004; Schlaggar and McCandliss, 2007; Shaywitz and Shaywitz, 2005, 2008] and quantitative meta‐analyses [e.g., Maisog et al., 2008; Richlan et al., 2009, 2011]. In the field of developmental dyslexia, quantitative coordinate‐based meta‐analyses were concerned with different indices of brain structure and function, such as task‐related dysfunctions as measured by fMRI and PET [Maisog et al., 2008; Richlan et al., 2009, 2011], local gray matter alterations as measured by voxel‐based morphometry [Linkersdörfer et al., 2012; Richlan et al., 2013], and white matter integrity as measured by diffusion tensor imaging [Vandermosten et al., 2012].

Meta‐analyses of reading‐related dysfunctions in dyslexia [Maisog et al., 2008; Richlan et al., 2009, 2011] identified left‐hemisphere regions with consistent functional brain abnormalities. Dyslexic readers exhibit lower activation relative to nonimpaired readers in left temporoparietal cortex (TPC), occipitotemporal cortex (OTC), and inferior frontal gyrus (IFG). In addition, dyslexics show consistently higher activation in the left precentral gyrus (PRG) [Richlan et al., 2009]. For reasons of simplicity, hereafter lower/higher activation in dyslexic compared with nonimpaired will be referred to as underactivation/overactivation.

What is missing so far is a quantitative meta‐analysis concerned with how the functional neuroanatomical signature of developmental dyslexia across alphabetic writing systems is influenced by orthographic depth. In particular, the question is whether different behavioral manifestations arising from the regularity of grapheme–phoneme relations in different languages are associated with different functional neuroanatomical manifestations. Recently, Richlan [2014] underscored the role of orthographic depth [e.g., Frost et al., 1987; Schmalz et al., 2015] in the functional neuroanatomy of developmental dyslexia.

Persistent slow and dysfluent word recognition is a hallmark of dyslexia across orthographies [e.g., Katzir et al., 2004], whereas inaccurate mapping from graphemes to the corresponding phonemes is characteristic for dyslexia in irregular or deep orthographies (DO), especially for English. This is evident by the (non)word reading accuracy advantage of dyslexic children learning to read in shallow orthographies (SO) compared with DO [Barca et al., 2006; Davies et al., 2007; Landerl et al., 1997; Landerl and Wimmer, 2000; Spinelli et al., 2005; Wimmer, 1993; Wimmer and Schurz, 2010; Ziegler et al., 2003; Zoccolotti et al., 2005].

The severe reading speed deficit together with poor spelling performance characterizes dyslexia in SO [Wimmer and Schurz, 2010]. The poor but phonetically acceptable spelling is explained by the fact that German (similar to other SO) is regular in the reading but not in the writing direction [e.g., Wimmer et al., 2010]. Poor spelling abilities provide evidence for an orthographic lexicon deficit, which results from insufficient stored representations of whole words or larger multiletter recognition units. According to the phonological deficit hypothesis, the primary problem of dyslexic readers has to do with phonological word decoding based on serial grapheme–phoneme conversion [e.g., Shaywitz and Shaywitz, 2005; Snowling, 2000; Vellutino and Fletcher, 2005].

To account for this inefficient access from orthography to phonology in SO, Wimmer [1993] adapted the dominant phonological deficit explanation and proposed a phonological speed deficit explanation [Wimmer and Schurz, 2010]. In this perspective, the reading speed impairment was suggested to reflect an orthographic lexicon deficit, as well as inefficient access to lexical (addressed) and sublexical (assembled) phonology [e.g., Bergmann and Wimmer, 2008]. Recent findings support this alternative hypothesis of a deficit in the access to otherwise intact phonological representations [Boets et al., 2013; Ramus, 2014].

The identification of universal and orthography‐specific dyslexic brain activation abnormalities has the potential to substantially improve and extend our neurobiological understanding of developmental dyslexia [Frost, 2012]. So far, most neurocognitive models of developmental dyslexia [e.g., Démonet et al., 2004; McCandliss and Noble, 2003; Pugh et al., 2001; Sandak et al., 2004] are based on findings from English‐language studies. In light of this Anglo‐centric research, Share [2008] pointed to the extreme ambiguity of the English spelling‐sound correspondence and critically emphasized the significant influence of this “outlier” orthography on previous theoretical conceptualizations of reading and dyslexia. Therefore, it is of specific interest whether these previous findings of functional brain abnormalities can be generalized to dyslexic readers of other, more regular orthographies. Besides the high relevance for the research domain, this topic is all the more relevant for practice. That is, different neurobiological manifestations of dyslexia in DO and SO would have an impact on the identification of at‐risk children, prevention, diagnosis, and remediation.

Cross‐language neuroimaging studies can directly address this question. Up to date, however, Paulesu et al. [2001] provided the first and only evidence from a cross‐linguistic brain imaging study on dyslexic dysfunctions in different alphabetic languages. They compared brain activation in nonimpaired and dyslexic Italian, French, and English university students during word and nonword reading by using PET. Relative to nonimpaired readers, dyslexic readers exhibited underactivation in superior, middle, and inferior temporal and occipital gyri regardless of orthography. Comparing the dyslexic subsamples from the three languages differing in orthographic depth, Paulesu et al. [2001] did not find orthography‐specific effects. Thus, the authors claimed that their findings speak for a universal neurocognitive basis of developmental dyslexia. For a detailed critical discussion of this study and for a review of other studies on the comparison to nonalphabetic writing systems, within‐subjects designs in bilinguals, and artificial language learning, see Richlan [2014].

Hadzibeganovic et al. [2010] questioned the biological unity of developmental dyslexia and criticized the methodological approach used by Paulesu et al. [2001]. Their critique concerns in particular the heterogeneity between the groups (formal diagnosis of dyslexia in English and French, but not in Italian participants). A recent review by Richlan [2014] also re‐examined the seminal work of Paulesu et al. [2001]. Richlan [2014] described in detail why the way Paulesu et al. [2001] searched for orthography‐specific effects was not optimal. Rather than comparing dyslexic activation directly across different orthographies, Richlan [2014] suggested that the comparison of the relative abnormalities of dyslexic readers (compared with nonimpaired readers) is more sensible.

Besides the only published cross‐language study, there is an increasing number of imaging studies on dyslexia conducted within a single alphabetic language. Even if orthography‐related differences are not the focus of these studies, meta‐analyses allow comparing brain dysfunction patterns of dyslexic readers in DO versus dyslexic readers in SO. In fact, a similar approach was recently used to identify shared and distinct reading‐related activation patterns in typical English compared with typical Dutch, Finnish, German, Italian, and Swedish readers [Martin et al., under review] and in child and adult readers [Martin et al., 2015]. In the domain of dyslexia, this meta‐analytic approach was used for the first time by Richlan et al. [2011]. The authors investigated age‐related differences in dyslexic brain activation abnormalities by comparing studies with dyslexic children versus nonimpaired children to studies of dyslexic adults versus nonimpaired adults.

The present coordinate‐based meta‐analysis aimed to clarify whether the functional neuroanatomical manifestation of dyslexia is similar in written alphabetic languages differing in orthographic depth. Thus, this study provides a novel approach in the systematic comparison of functional brain abnormalities across alphabetic languages. We selected one set of neuroimaging studies conducted in a DO (English) and a second set of studies conducted in SO (Dutch, German, Italian, and Swedish) [Borgwaldt et al., 2005; Seymour et al., 2003]. To identify and localize universal reading‐related brain activation abnormalities in dyslexia, separate meta‐analyses were computed for the two orthography‐specific sets. To further identify orthography‐specific abnormalities, these separate maps were directly compared in a meta‐analytic difference map. This procedure was already used in former studies [Martin et al., under review; Martin et al., 2015; Richlan et al., 2009, 2011, 2013; Schurz et al., 2014b].

In general, we expected dyslexic under‐ and overactivation within the same core reading network in DO and SO, but depending on differential weighing of cognitive processes, with a different degree and spatial extent of the local clusters [see also Pugh et al., 2005]. In other words, there might be universal dysfunctions together with orthography‐specific effects depending on particular characteristics and processing demands of the language.

In line with the classical phonological deficit hypothesis, one would expect a primary dysfunction in the left TPC, accompanied by a secondary dysfunction in the left OTC [e.g., Pugh et al., 2000]. This expectation is independent of the orthography. In accordance with orthography‐specific findings from typical readers [Paulesu et al., 2000], the expectations would be different. Given that typical readers in DO primarily use an addressed reading strategy (lexical encoding via access to stored visual orthographic representations, retrieval from the mental lexicon), one would expect more pronounced underactivation in brain regions associated with whole‐word recognition (e.g., the VWFA in the left ventral OTC) in dyslexic readers of DO. In contrast, one would expect underactivation in brain regions associated with phonological processing (e.g., the posterior STG in the left TPC) predominantly in dyslexic readers of SO because typical readers can rely on a phonology‐based assembled reading strategy (sublexical encoding via serial grapheme–phoneme conversion).

Taking into account that dyslexic readers in SO suffer from a phonological speed deficit, one would expect a primary dysfunction in the left OTC. In particular, the impaired reading speed associated with an insufficient orthographic lexicon together with inefficient access to lexical and sublexical phonology [e.g., Richlan et al., 2010; Richlan, 2012; Wimmer et al., 2010] should result in underactivation in the VWFA of the left OTC [Dehaene and Cohen, 2011]. Assuming the left OTC functions as an interface to phonology [Price and Devlin, 2011], this area is presumably the best candidate for a universal effect of the common visual–verbal speed deficit.

With respect to dyslexic overactivation, there are no indications of orthography‐related differences so far. Therefore, we expected overactivation in the left PRG and right hemisphere regions regardless of orthography because of universal overreliance on articulatory processes. Given that dyslexic overactivation was previously associated with compensatory mechanisms, possible differences would be of special interest.

## MATERIALS AND METHODS

For the present coordinate‐based meta‐analysis, we selected studies according to previous meta‐analyses [Richlan et al., 2009, 2011]. Several Medline/PubMed searches (http://www.pubmed.org) with the keywords “dyslexia” and “brain imaging,” “fMRI,” “functional magnetic resonance imaging,” “PET,” “Positron Emission Tomography,” “neuroimaging,” “functional abnormalities,” “brain abnormalities,” or “dysfunction” were performed to select fMRI and PET studies that met the following criteria: (1) visual stimuli were letter strings of words or nonwords, (2) tasks were reading or reading‐related (e.g., rhyme judgments, phonological lexical decision), and (3) whole‐brain analyses of group comparisons (dyslexic vs nonimpaired readers) were reported in a standard stereotactic space (Talairach or MNI). Furthermore, to increase homogeneity, we restricted the study selection to studies of alphabetic writing systems.

### Deep Orthographies

On the basis of these criteria, we identified 13 studies with English‐speaking participants as suitable for inclusion in meta‐analyses on dyslexic brain activation abnormalities in DO. Tanaka et al. [2011] used two independent samples investigated at two sites, Carnegie Mellon University and Stanford University. Since group comparisons were reported separately for each sample, we included both samples. In the following, we refer to the 14 included samples for DO studies as 14 studies for reasons of simplicity.

### Shallow Orthographies

We used additional keywords “orthography,” “shallow,” “transparent,” “Dutch,” “Finnish,” “German,” “Italian,” “Polish,” “Spanish,” or “Swedish,” to find studies investigating dyslexic brain activation abnormalities in SO. On the basis of the aforementioned criteria, we identified 14 studies as suitable for inclusion in meta‐analyses on dyslexic dysfunctions in SO: German (11), Italian (2), and Swedish (1). From a large (so far unpublished) multicenter study in which our lab was involved [Maurer et al., under review], we included the data from the Dutch, German, and Swiss‐German extreme dyslexics (<5th percentile) and good readers (>50th percentile). Two studies—an English [Meyler et al., 2007] and a German [Schulz et al., 2008]—were excluded in favor of more recent studies with the same participants [Meyler et al., 2008; Schulz et al., 2009]. The study by Paulesu et al. [2001] who examined English, French, and Italian dyslexics was excluded in favor of Brunswick et al. [1999] who reported group differences between dyslexic and nonimpaired readers separately for the English subsample.

In sum, 28 studies (23 fMRI and 5 PET) with a total number of 435 participants from DO studies (232 dyslexics and 203 controls) and 472 participants from SO studies (219 dyslexics and 253 controls) were included in the present meta‐analysis.

To ensure statistical independence, we included for each study only foci of group differences from a single contrast. In addition, we balanced the number of contrasts for word and nonword reading across both sets of studies to increase comparability. For Hoeft et al. [2006] and Schulz et al. [2009], we used the comparison with the age‐matched and not with the reading‐level‐matched controls. Several potentially relevant studies were not included because they did not report direct group comparisons for baseline contrasts in a standard stereotactic space on the whole‐brain level [e.g., Aylward et al., 2003; Backes et al., 2002; Rimrodt et al., 2009; Shaywitz et al., 2007].

The selected studies and their main characteristics are listed in Table 1. Altogether we included 73 foci of underactivation from 14 DO studies and 73 foci of underactivation from 14 SO studies. In addition, we included 22 foci of overactivation from 8 (out of the 14) DO studies and 86 foci of overactivation from 9 (out of the 14) SO studies. Given this imbalance, the direct comparison between deep and SO with respect to dyslexic overactivation has to be interpreted cautiously. The inclusion of foci of overactivation in the meta‐analysis, however, increases the accuracy of the individual study‐specific maps and allows a complete assessment of the neuroanatomical manifestation of dyslexic functional brain abnormalities.

**Table 1:** Main characteristics of the included fMRI studies and number of foci used in the meta‐analysis

Pair	Year	First author	Imaging	*N*	Dys	Con	Native language	Age mean (SD; range)	Task type	Contrast	Threshold		No. of foci under‐/overactivation^a^
											Voxel‐level *p* <	Cluster‐level *p* < or no. of voxels	
*Deep orthographies*	2007	Booth	fMRI	26	13	13	English	10.5 (2.3)	Semantic relationship judgment	Related W pairs > fixation	0.001 unc.	15 voxels	1(0)/1
	1999	Brunswick	PET	12	6	6	English	23.1 (4.1)	Reading aloud	W & NW > rest	0.001 unc.	–	14(0)/1
	2006	Cao	fMRI	28	14	14	English	11.6 (8–14)	Word rhyme judgment	W conflicting trials > fixation	0.001 unc.	15 voxels	6(0)/0
	2006	Hoeft	fMRI	20	10	10	English	11.2 (0.5)	Word rhyme judgment	W rhyming > fixation (age‐matched)	0.001 unc.	10 voxels	6(6)/0
	2007	Hoeft	fMRI	38	19	19	English	14.4 (2.2)	Word rhyme judgment	W rhyming >fixation (age‐matched)	0.001 unc.	10 voxels	4(4)/7
	2010	Hu	fMRI	21	11	10	English	13.7 (12–16)	Semantic relationship judgment	W matching > fixation	0.001 unc.	0.05 FWE	6(0)/1
	2010	Landi	fMRI	26	13	13	English	13.2 (9–19)	Word and nonword rhyme judgment	NW rhyming > baseline	0.01 FDR	20 voxels	0(0)/2
	2005	McCrory	PET	18	8	10	English	20.2 (1.9)	Reading aloud	W reading > false fonts	0.05 FWE	–	1(0)/0
	2008	Meyler	fMRI	35	23	12	English	10.8 (0.5)	Sentence comprehension	Sentence reading > fixation (pre‐remediation)	0.002 unc.	10 voxels	6(6)/2
	1996	Paulesu	PET	10	5	5	English	26.2 (1.9)	Letter pair rhyme judgment	Letter pair rhyming > shape similarity judgment	0.001 unc.	–	6(0)/0
	1997	Rumsey	PET	31	17	14	English	26.0 (6.5)	Reading aloud	NW > fixation	0.001 unc.	9 voxels	14(8)/7
	2011a	Tanaka	fMRI	57	31	26	English	10.3 (1.1)	Word rhyme judgment	W rhyming > fixation	0.05 FDR	–	2(1)/0
	2011b	Tanaka	fMRI	74	38	36	English	13.4 (2.5)	Word rhyme judgment	W rhyming > fixation	0.05 FDR	–	2(1)/0
	2001	Temple	fMRI	39	24	15	English	10.6 (1.4)	Letter matching	Matching letters > matching lines	0.001 unc.	20 voxels	5(0)/1
*Shallow orthographies*	2010	Bach	fMRI	32	14	18	(Swiss‐) German	8.3 (0.4)	Word and nonword letter substitution and lexical decision	W & NW substitution > rest	0.005 unc.	24 voxels	0(0)/14
	2006	Brambati	fMRI	24	13	11	Italian	30.5 (13–63)	Silent reading	W & NW reading > false fonts	0.05 unc.	20 voxels	9(0)/0
	1999	Georgiewa	fMRI	34	17	17	German	14.0 (9–17)	Silent reading	NW reading > false fonts	0.05 unc.	0.05 unc.	2(0)/2
	2004	Grünling	fMRI	38	17	21	German	13.6 (1.4)	Nonword rhyme judgment	NW rhyming > letter string judgment	0.01 unc.	10 voxels	3(0)/31
	2002	Ingvar	PET	18	9	9	Swedish	23.5 (20–28)	Silent reading	W reading > rest	0.001 unc.	–	3(0)/3
	2006	Kronbichler	fMRI	28	13	15	German	15.7 (0.7)	Sentence comprehension	Sentence reading > false fonts	0.01 FDR	4 voxels	2(0)/13
	2013	Kronschnabel	fMRI	35	13	22	(Swiss‐) German	16.0 (0.6)	Hash detection	W > rest	0.005 unc.	160 voxels	4(0)/0
	2011	Maurer	fMRI	27	11	16	(Swiss‐) German	11.4 (0.4)	One‐back	W > rest	0.01 unc.	30 voxels	13(0)/1
	under review	Maurer	fMRI	66	31	35	Dutch, (Swiss‐) German	10.0 (1.3)	Semantic decision (animal/object)	W > symbol strings	0.01 unc.	34 voxels	12(0)/8
	2011	Pecini	fMRI	26	13	13	Italian	23.0 (13.0)	Word rhyme generation	W rhyme generation > rest	0.05 corr.	4 voxels	4(0)/0
	2010	Richlan	fMRI	33	15	18	German	18.0 (1.1)	Phonological lexical decision	W > fixation	0.005 unc.	20 voxels	3(0)/6
	2009	Schulz	fMRI	30	15	15	German	11.5 (0.4)	Sentence comprehension	Sentence reading > fixation (age‐matched)	0.001 unc.	5 voxels	9(3)/0
	2011	Van der Mark	fMRI	42	18	24	(Swiss‐) German	11.4 (0.6)	Phonological lexical decision	Pseudohomophones > fixation	0.001 unc.	10 voxels	8(0)/0
	2010	Wimmer	fMRI	39	20	19	German	20.6 (6.8)	Phonological lexical decision	W > fixation	0.005 unc.	10 voxels	1(0)/8

a Foci of underactivation resulting from deactivations are reported in parentheses.

In Table 1, it is specified how many foci of underactivation and overactivation were extracted from each study. Some of the studies reported foci of dyslexic underactivation that resulted from deactivation relative to baseline (listed in Table 1 in parentheses). That is, dyslexic readers exhibited stronger task‐negative activation compared with typical readers. Otherwise, underactivation means that task‐positive activation is lower or absent in dyslexics compared with controls. It is worth noting that not all the included studies provided information about this distinction. One study explicitly excluded foci of deactivation [Richlan et al., 2010]. As a former meta‐analytic finding of underactivation in the inferior parietal lobule (IPL) was driven by deactivation foci [Richlan et al., 2011], we checked the influence of deactivation foci on the present results. Therefore, we repeated the separate meta‐analyses for DO and SO studies and the statistical comparison with all deactivation foci excluded.

### Meta‐Analytic Method

For the present coordinate‐based meta‐analysis, we used Seed‐based *d* Mapping (SDM; formerly Signed Differential Mapping) software (http://www.sdmproject.com), version 4.31 [Radua et al., 2010, 2012, 2014; Radua and Mataix‐Cols, 2009]. Based on reported foci of under‐ and overactivation, their respective statistical values, and the sample size, SDM recreates maps of effect‐sizes (Hedge's *d*) for each original study. Thereby, anisotropic kernels are used to account for spatial anisotropy of activation clusters due to anatomical constraints.

All foci of under‐ and overactivation reported by the individual studies were transformed to MNI space with a built‐in feature using the icbm2tal transform [Lancaster et al., 2007]. Meta‐analyses were restricted to a specific gray‐matter template provided by the software. Effect size maps were recreated for each study by convolving reported foci with a fully anisotropic un‐normalized Gaussian kernel (*α* = 1). The anisotropy of the kernel was based on the spatial correlations of the gray‐matter template. Within a study, values obtained by close anisotropic kernels were combined by square‐distance‐weighted averaging. To combine the data across study‐specific effect size maps, a random effects general linear model was used. Statistical significance was examined by a permutation test that randomizes the location of activation foci within the SDM gray‐matter template (500 randomizations). The meta‐analytic maps were thresholded using the recommended voxel‐level (height) threshold of *p* < 0.005 (uncorrected) and a cluster‐level (extent) threshold of 10 voxels. This uncorrected threshold was recommended for SDM as it was found to optimally balance sensitivity and specificity, and to be an approximate equivalent to a corrected threshold of *p* < 0.05 in original neuroimaging studies [Radua et al., 2012].

To investigate regions of convergent under‐ and overactivation across studies in each orthography‐specific set, we computed two separate meta‐analytic maps. We determined universal effects by overlaying the two separate maps onto a single‐template brain and identifying regions of overlapping activation. Furthermore, a linear model analysis was used to compare the two sets of studies. The resulting difference map was thresholded with the same parameters as the separate maps. This direct comparison of dyslexic brain abnormalities in DO and SO informs on statistically reliable orthography‐specific effects.

We used systematic whole‐brain voxel‐based jackknife sensitivity analysis to evaluate the replicability of the meta‐analytic findings. That is, the separate meta‐analyses were repeated for the number of included studies while excluding each time a different study (i.e., the separate meta‐analyses were repeated 14 times, with a different combination of 13 included studies each). The rationale behind this procedure is that if a meta‐analytic finding remains statistically significant in all or most of the combinations of studies, it can be concluded that this finding is robust against changes of the sample and thus highly replicable [Radua and Mataix‐Cols, 2009].

For further evaluation of the robustness of the meta‐analytic findings, we inspected how many of the original studies contributed to the identification of each meta‐analytic cluster. Therefore, we checked whether the reported coordinates fell within a 20 mm sphere around the peak of the identified meta‐analytic clusters. In addition, we used the SPM Anatomy toolbox [Eickhoff et al., 2007, 2005] to ensure that the input focus can be assigned to the same anatomical substrate. This method provides a straightforward assessment of the consistency of meta‐analytic findings and was already used in former meta‐analyses from our lab [Martin et al., 2015; Richlan et al., 2009, 2011, 2013].

## RESULTS

**Figure 1:**
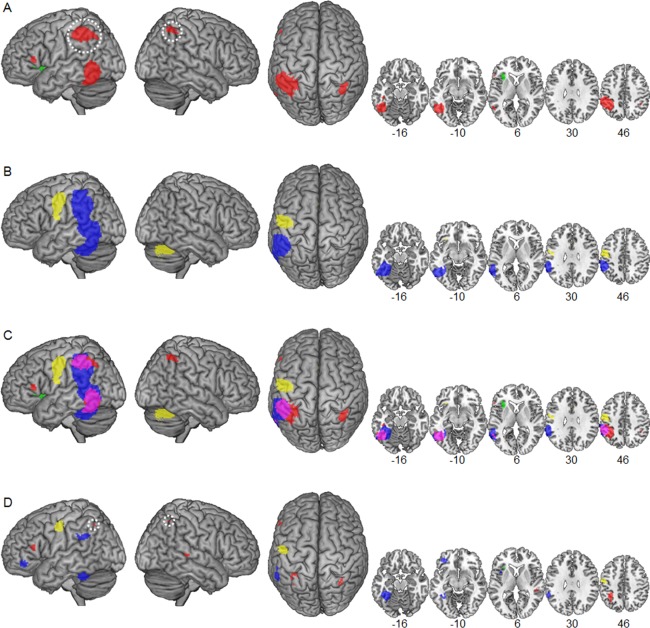
Surface renders and selected slices illustrating convergent dyslexic underactivation (red) and overactivation (green) in deep (A) and convergent (blue) dyslexic underactivation and overactivation (yellow) in shallow orthographies (B) identified in the present meta‐analysis. White circles indicate the disappearance of the left IPL and right IPS abnormalities in deep orthographies after exclusion of deactivation foci. (C) The overlay of the two separate maps for deep and shallow orthographies illustrates overlapping underactivation (purple). The difference map in (D) indicates higher convergence of underactivation for deep compared with shallow (red) and vice versa (blue) and higher convergence of overactivation for deep compared with shallow (green) and vice versa (yellow). [Color figure can be viewed in the online issue, which is available at http://wileyonlinelibrary.com.]

The pattern of functional brain abnormalities identified in the present meta‐analysis is shown in Figure 1 (rendered on a template brain). Table 2 shows the clusters of convergent reading‐related dyslexic under‐ and overactivation characterized by the MNI coordinates, the SDM‐*Z* values of local maxima, and the extent of the clusters.

**Table 2:** Results of the separate meta‐analyses of dyslexic functional brain abnormalities in deep and shallow orthographies

Region	MNI co‐ordinates			SDM‐*Z*	Voxels	JK
	*x*	*y*	*z*			
Deep orthographies						
*Underactivation*						
L inferior parietal lobule^a^	−40	−42	46	−2.99	1626	14
L occipitotemporal cortex	−50	−58	−10	−2.39	1561	14
R intraparietal sulcus^a^	36	−50	50	−1.59	150	12
L optic radiation	−26	−70	2	−1.86	51	11
L inferior frontal gyrus, pars triangularis	−56	26	10	−1.48	34	12
*Overactivation*						
L anterior insula	−32	26	4	1.30	286	13
Shallow orthographies						
*Underactivation*						
L occipitotemporoparietal cortex					5450	14
L fusiform gyrus	−42	−42	−18	−4.85		
L inferior parietal lobule	−54	−38	40	−2.62		
L inferior temporal gyrus	−58	−60	−8	−2.51		
L middle temporal gyrus	−60	−50	0	−2.26		
*Overactivation*						
L precentral gyrus	−52	−8	44	2.50	853	14
R cerebellum	30	−66	−30	1.95	904	13
L caudate	−12	0	18	1.69	117	12
L inferior frontal gyrus, pars orbitalis	−32	26	−10	1.53	16	12
R caudate	14	14	14	1.44	50	12
R inferior frontal sulcus	36	20	24	1.42	12	2
L cingulate gyrus	−4	20	22	1.40	25	11

a Underactivation resulting from increased deactivation relative to baseline (see Discussion).

L = left, R = right, JK = jackknife analysis (number of subsamples that replicate the finding).

### Separate Maps

For DO, the meta‐analysis identified five clusters with convergent underactivation (Fig. 1A marked in red and Table 2) in left dorsal IPL, left OTC, right intraparietal sulcus (IPS), left optic radiation, and left IFG pars triangularis (in order of spatial extent). Convergent overactivation in DO (Fig. 1B marked in green) was identified in the left anterior insula.

For SO, the meta‐analysis identified one large cluster with convergent underactivation (Fig. 1B marked in blue and Table 2) in widespread left occipitotemporoparietal regions. This substantial cluster included dorsally a maximum in the dorsal IPL and ventrally maxima in fusiform, inferior, and middle temporal gyri (FFG, ITG, and MTG, respectively). The cluster extended in the parietal cortex into the left supramarginal gyrus (SMG), in the temporal cortex into the superior temporal gyrus (STG), and inferior into the cerebellum.

Convergent overactivation in SO (Fig. 1B marked in yellow) was identified in the left PRG, the right cerebellum, bilateral caudate nuclei, left IFG pars orbitalis, right inferior frontal sulcus (IFS), and left cingulate gyrus.

White circles in Figure 1A indicate the disappearance of the bilateral IPL underactivation for DO after exclusion of foci identified in the original studies with deactivation relative to baseline. As there may be different functional roles of increased task‐negative activation compared with reduced task‐positive activation (see Discussion), we repeated the separate meta‐analyses for DO and SO studies and the statistical comparison with all deactivation foci excluded. While the abnormalities in the IPL disappeared from the map of DO studies, all other originally identified clusters with underactivation were nearly unaffected with identical localization of maxima and only slightly changed extents.

### Robustness of Meta‐Analytic Findings

Tables III and IV show which of the original studies reported one or more foci contributing to the identified meta‐analytic under‐ and overactivation clusters. Additional results of the individual studies that found no support in the present meta‐analyses are reported in the rightmost column of Tables III and IV, respectively (overactivations in italics). Because of little consistency across studies, these regions did not result in statistically significant meta‐analytic clusters.

**Table 3:** Convergence across studies in deep orthographies

		Underactivation						
Year	First author	L inferior parietal lobule	L occipito‐temporal cortex	R intra‐parietal sulcus	L optic radiation	L inferior frontal gyrus	Overactivation L anterior insula	Additional regions
2007	Booth					X		*R SMG*
1999	Brunswick		X					L LG, CB, caudate, *L PRG*
2006	Cao	X	X			X		L PRG, R MFG
2006	Hoeft	X D		X	X			L MFG, R MTG, SFG
2007	Hoeft	X D	X D		X D		X	R FFG, *L MFG, R Th*
2010	Hu	X	X		X	X		L MFG, CB, R PRG, *L PRG*
2010	Landi							*R PRG, MTG*
2005	McCrory		X					
2008	Meyler	X D		X				L MFG, SPL, *B SMA*
1996	Paulesu							L MFG, STG, insula, R SMA, striatum
1997	Rumsey	X D	X D				X	L SPL, B SMG, STG, R FFG, IPL, PC, *L FFG, PRG, Th, CB, R insula*
2011a	Tanaka	X D	X					
2011b	Tanaka	X D	X					
2001	Temple			X				L MOG, PC, B CG, *L IFG*
								
	Total	8 (6)	8 (2)	3	3 (1)	3	2	

For each region identified with functional abnormalities, studies reporting one or more foci in this region are marked with an X. In addition, underactivation resulting from increased deactivation relative to baseline is marked with a D. Furthermore, findings of functional abnormalities in additional brain regions are reported (overactivation in italics).

B = bilateral, CB = cerebellum, CG = cingulate gyrus, FFG = fusiform gyrus, IFG = inferior frontal gyrus, IPL = inferior parietal lobule, L = left, LG = lingual gyrus, MFG = middle frontal gyrus, MOG = middle occipital gyrus, MTG = middle temporal gyrus, PC = precuneus, PRG = precentral gyrus, R = right, SFG = superior frontal gyrus, SMA = supplementary motor area, SMG = supramarginal gyrus, SPL = superior parietal lobule, STG = superior temporal gyrus, Th = thalamus.

**Table 4:** Convergence across studies in shallow orthographies

Year	First author	Underactivation		Overactivation						Additional regions
		L inferior parietal lobule	L occipito‐temporal cortex	L precentral gyrus	R cerebellum	B caudate nucleus	L inferior frontal gyrus	R inferior frontal sulcus	L cingulate gyrus	
2010	Bach			X				X		*B STG, R SFG, PRG, SPL, PC*
2006	Brambati	X	X							L STG^a^, R MFG, CB
1999	Georgiewa									L Th, *L STG, R HG*
2004	Grünling			X	X	X	X	X	X	R SFG, *L PC, MFG, B SFG, MTG, R IFG, LG, insula, CG*
2002	Ingvar									R MFG, SFG, AG, *R MTG, optic radiation*
2006	Kronbichler	X	X	X	X	X	X			*L MTG, B LG, Th*
2013	Kronschnabel		X							R MFG, IOG
2011	Maurer	X	X							R SFG, L MFG, IOG, PC, *R CG*
Under review	Maurer	X	X	X	X		X			L SFG, B MFG, R ITG, *L PC*
2011	Pecini	X								L STG^a^, MFG, PC
2010	Richlan		X		X	X				R IOG, *L LG, SMA, Th*
2009	Schulz	X D	X							L MTG^a^, SFG, MFG
2009	Van der Mark	X	X							B insula, MFG, R POG, SMG, IPL
2010	Wimmer		X	X		X			X	*R calcarine, SFG, CG*
	Total	7(1)	9	5	4	4	3	2	2	

For each region identified with functional abnormalities, studies reporting one or more foci in this region are marked with an X. In addition, underactivation resulting from increased deactivations relative to baseline is marked with a D. Furthermore, findings of functional abnormalities in additional brain regions are reported (overactivation in italics).

a Reported foci that contribute to the temporal portion of the large left occipitotemporoparietal cluster (see Results section).

AG = angular gyrus, B = bilateral, CB = cerebellum, CG = cingulate gyrus, HG = Heschl's gyrus, IFG = inferior frontal gyrus, IOG = inferior occipital gyrus, IPL = inferior parietal lobule, ITG = inferior temporal gyrus, L = left, LG = lingual gyrus, MFG = middle frontal gyrus, MTG = middle temporal gyrus, PC = precuneus, POG = postcentral gyrus, PRG = precentral gyrus, R = right, SFG = superior frontal gyrus, SMA = supplementary motor area, SMG = supramarginal gyrus, SPL = superior parietal lobule, STG = superior temporal gyrus, Th = thalamus.

Table 3 shows the high convergence across DO studies for left OTC and left dorsal IPL underactivation with 8 (out of 14) studies contributing each. One of the 14 included DO studies did not report any foci of dyslexic underactivation [Landi et al., 2010]. Only limited convergence was found for the right IPS, left optic radiation, and left IFG pars triangularis underactivation with 3 studies contributing each. Additionally, limited convergence across studies was found for left insula overactivation, with only 2 (out of 8) studies contributing. For 6 of the 14 included DO studies, no foci of dyslexic overactivation were reported (Table 1).

Table 4 shows the substantial convergence across SO studies for the large underactivation cluster that extended into occipital, temporal, and parietal regions. To increase regional specificity and to facilitate the comparison with the meta‐analysis of DO studies, this widespread cluster was divided into a ventral section in the left OTC and a dorsal section in the left IPL. Nine studies contributed to the left OTC and 7 studies contributed to the left IPL. One of the 14 included SO studies did not report any foci of dyslexic underactivation [Bach et al., 2010]. For the left PRG overactivation cluster, high convergence across SO studies was found with 5 out of 9 studies contributing. Five studies did not report any foci of dyslexic overactivation (Table 1). Limited convergence was found for overactivation in the right cerebellum and bilateral caudate nuclei with 4 each and in the left IFG pars orbitalis with 3 studies contributing. The remaining meta‐analytic overactivation clusters in cingulate gyrus and right IFS showed low convergence across studies with only 2 studies contributing.

Tables III and IV further indicate the contribution of deactivation foci to the identified regions (marked with a D). A remarkable finding is that 6 out of 8 DO studies contributed with deactivation foci to left dorsal IPL cluster. In contrast, only 1 (out of 7) SO study contributed with deactivation foci to the IPL cluster identified for SO. After exclusion of the deactivation foci from our meta‐analysis, the dorsal left IPL and right IPS underactivation clusters disappeared from the map of DO (indicated by white circles in Fig. 1A; for a similar finding, see Richlan et al. [2011]). All other originally identified regions with underactivation were nearly unaffected with identical localization of maxima and only slightly changed extents.

### Replicability of Meta‐Analytic Findings

For evaluation of the replicability, we used jackknife sensitivity analysis. Table 2 shows that the main meta‐analytic findings remained unchanged in most recalculations of the meta‐analysis, indicating robustness against changes of the samples. For the meta‐analysis of DO studies, we found perfect replicability of underactivation in the left dorsal IPL and left OTC (14 out of 14 leave‐one‐out recalculations) and high replicability of underactivation in the right IPS (12), left IFG (12), and left optic radiation (11). The left insula overactivation was identified in 13 out of 14 leave‐one‐out recalculations.

Similarly, for the meta‐analysis of SO studies, findings were perfectly replicable for the left dorsal IPL and left OTC parts of the large left occipitotemporoparietal underactivation cluster (14 out of 14 leave‐one‐out recalculations, respectively). Similarly, the finding of overactivation in the left PRG was perfectly replicable (14). We found high replicability of overactivation in right cerebellar (13), right and left caudate (12 each), and left cingulate gyrus (11). The right IFS overactivation was identified with very low replicability with only 2 out of 14 leave‐one‐out recalculations.

### Overlap

For the identification of overlapping regions of dyslexic functional abnormalities in DO and SO, Figure 1C shows the separate meta‐analytic maps rendered on a template brain. An overlapping region of underactivation in DO and SO (marked in purple) was identified in the left OTC. This overlap extended from anterior to posterior OTC (*x* = −63 to −36, *y* = −70 to −29, *z* = −26 to 9) including regions in FFG, ITG, MTG, and inferior occipital gyrus (IOG). Convergent underactivation in SO extended to temporal and parietal regions superior to the left OTC overlap (above *z* = 10). Furthermore, it extended medial (*x* = −35 to −21) and inferior into the left cerebellum (*z* = −27 to −37). In contrast, convergent underactivation in DO extended to the ventral ITG anterior to the overlap (*y* = −28 to −19).

In addition, an overlap between underactivation in DO and SO was localized in the left dorsal IPL (*x* = −63 to −40, *y* = −27 to −56, *z* = 35–57). With respect to this left dorsal IPL cluster, convergent underactivation in DO (Fig. 1C, marked in red) extended more medially (*x* = −39 to −25) and posterior (*y* = −57 to −66). In contrast, convergent underactivation in SO (Fig. 1C, marked in blue) reached more inferior (below *z* = 34) into the left ventral SMG and posterior STG. The overlap in the left IPL was no longer present when foci resulting from deactivation were excluded from the meta‐analysis as the IPL abnormality disappeared from the separate map of DO studies (indicated by a white circle in Fig. 1A).

### Difference Map

To obtain statistically reliable information on orthography‐specific differences, we directly compared the SO and DO studies in a meta‐analytic difference map. This difference map is illustrated in Figure 1D (rendered on a template brain). See Table 5 for brain regions with higher convergence of under‐/overactivation for DO compared with SO and vice versa.

**Table 5:** Results of the direct statistical comparison of functional brain abnormalities in deep and shallow orthographies

Region	MNI coordinates			SDM‐*Z*	Voxels
	*x*	*y*	*z*		
Higher convergence for DO > SO					
*Underactivation*					
L intraparietal sulcus^a^	−34	−46	42	1.76	232
R superior temporal sulcus	50	−32	4	1.36	51
L precuneus	−8	−74	34	1.35	26
L inferior frontal gyrus, pars triangularis	−56	28	12	1.38	19
R intraparietal sulcus^a^	32	−58	52	1.38	20
*Overactivation*					
L anterior insula	−32	24	8	1.00	13
Higher convergence for SO > DO					
*Underactivation*					
L fusiform gyrus	−40	−42	−16	2.44	937
L inferior frontal gyrus, pars orbitalis	−36	40	−8	1.18	126
L temporoparietal cortex	−58	−44	30	1.11	102
L frontal operculum cortex	−40	12	4	1.09	17
*Overactivation*					
L precentral gyrus	−54	−8	46	1.61	174

a Underactivation resulting from increased deactivation relative to baseline (see Discussion).

L = left, R = right.

The direct statistical comparison identified five clusters with higher convergence of underactivation in DO compared with SO (Fig. 1D, marked in red) centered in the left IPS (*x* = −34, *y* = −46, *z* = 42), right STS (*x* = 50, *y* = −32, *z* = 4), left precuneus (*x* = −8, *y* = −74, *z* = 34), left IFG pars triangularis (*x* = −56, *y* = 28, *z* = 12), and right IPS (*x* = 32, *y* = −58, *z* = 52), respectively. Higher convergence of overactivation for DO compared with SO (Fig. 1D axial slice *x* = 6, marked in green) was identified in a small cluster in the left anterior insula (*x* = −32, *y* = 24, *z* = 8). The difference in the bilateral IPS was no longer present after exclusion of deactivation foci.

Higher convergence of underactivation for SO compared with DO (Fig. 1D, marked in blue) was identified in the left FFG (*x* = −40, *y* = −42, *z* = −16), left IFG pars orbitalis (*x* = −36, *y* = 40, *z* = −8), left TPC (*x* = −58, *y* = −44, *z* = 30), and left frontal operculum cortex (*x* = −‐40, *y* = 12, *z* = 4). Importantly, the direct statistical comparison of the additional analysis with all deactivation foci excluded identified higher convergence of underactivation for SO compared with DO in the left ventral and dorsal IPL. This cluster was then significantly extended (1153 voxels) with a maximum located more superior (*x* = −54, *y* = −40, *x* = 40). Higher convergence of overactivation in SO compared with DO (Fig. 1D, marked in yellow) was identified in the left PRG (*x* = −54, *y* = −8, *z* = 46).

## DISCUSSION

The present coordinate‐based meta‐analysis was aimed at the identification of universal and orthography‐specific dyslexic brain activation abnormalities in deep (English) and shallow (Dutch, German, Italian, Swedish) orthographies. So far, developmental dyslexia was associated with a dysfunction of three key regions in the left hemisphere reading network, that is, the OTC, TPC/IPL, and IFG.

It is plausible to assume a universal neurobiological origin of dyslexia because of shared cognitive dysfunctions. In this view, the common reading speed impairment, due to an insufficient orthographic lexicon and inefficient access to lexical and sublexical phonology [e.g., Richlan, 2012], is linked to a—presumably universal—left OTC dysfunction [e.g., Pugh, 2006]. In addition, the different behavioral manifestation of dyslexia across DO and SO suggests different cognitive dysfunctions. That is, dyslexic readers in DO suffer from slow, effortful, and especially inaccurate reading [e.g., Landerl et al., 1997; Ziegler et al., 2003], whereas dyslexic readers in SO primarily suffer from slow and effortful reading [e.g., Wimmer, 1993; Zoccolotti et al., 1999]. This indicates an involvement of different cognitive components. Assuming further a differential weighing of cognitive processes (e.g., whole‐word‐recognition or serial grapheme–phoneme conversion), and hence different brain activation between typical readers of DO and SO, different brain activation abnormalities in dyslexia are expected.

Based on the different behavioral manifestation of dyslexia, also the diagnostic criteria for identification of dyslexic readers differ across DO and SO. The main diagnostic criteria used in the studies included in the present meta‐analysis are listed in Supporting Information. Possible implications of the different diagnostic criteria for the interpretation of converging and diverging findings between DO and SO are discussed in more detail in the “Limitations” section.

In line with the idea of a universal neurobiological origin of developmental dyslexia [e.g., Pugh, 2006], presumably associated with the common speed impairment in dyslexic readers across DO and SO, we found common underactivation in dyslexic compared with nonimpaired readers in left middle, inferior temporal, and occipitotemporal regions.

Differences between DO and SO studies were evident with respect to the degree, spatial extent, and exact anatomical location of the under‐ and overactivation clusters. Higher meta‐analytic convergence of underactivation in DO compared with SO studies was identified in bilateral IPS, right STS, left precuneus, and left IFG pars triangularis, whereas higher meta‐analytic convergence of underactivation in SO compared with DO studies was found in the left FFG, TPC, IFG pars orbitalis, and frontal operculum. Higher meta‐analytic convergence of overactivation in DO compared with SO was found in the left anterior insula, whereas higher meta‐analytic convergence of overactivation in SO compared with DO studies was found in the left PRG.

In the following, we will describe the meta‐analytic findings of brain abnormalities in dyslexic readers in DO and SO and discuss universal and orthography‐specific effects in the framework of current functional neuroanatomical models. From here on, we use the term under‐/overactivation when we refer to under‐/overactivation relative to nonimpaired readers for reasons of simplicity.

**Figure 2:**
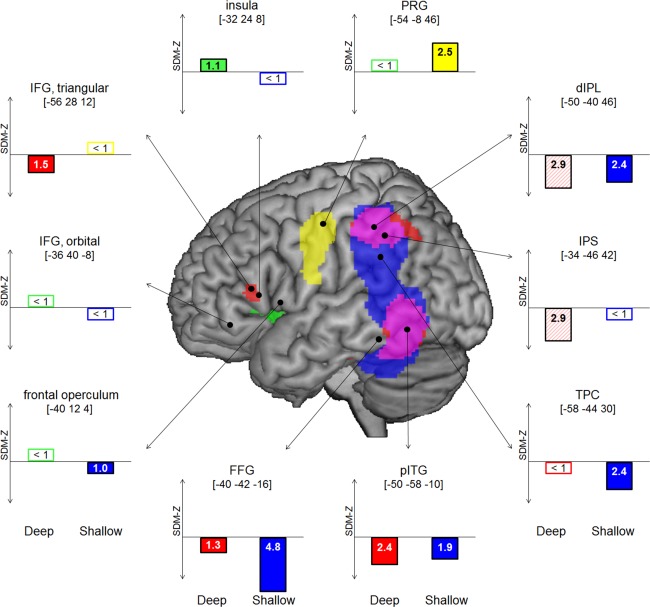
Regions of interest (ROI) in the left hemisphere illustrated on an overlay of the two separate maps for deep (underactivation: red, overactivation: green) and shallow (underactivation: blue, overactivation: yellow) orthographies. Common underactivation is illustrated in purple (Fig. 1C). The bar plots represent the SDM‐*Z* values of reading‐related under‐ and overactivation. Values above the statistical threshold (*p* < 0.005) are indicated by filled bars; values below the statistical threshold are indicated by outlined bars. Striped bars indicate the disappearance of the IPL abnormalities in deep orthographies after exclusion of deactivation foci. dIPL = dorsal inferior parietal lobule; FFG = fusiform gyrus; IFG = inferior frontal gyrus; IPS = intraparietal sulcus; pITG = posterior inferior temporal gyrus; PRG = precentral gyrus; TPC = temporoparietal cortex. [Color figure can be viewed in the online issue, which is available at http://wileyonlinelibrary.com.]

Figure 2 summarizes the main findings of the present meta‐analysis. Bar plots of meta‐analytic SDM‐*Z* values are displayed for selected regions that exhibited either universal or orthography‐specific brain abnormalities in dyslexic compared with nonimpaired readers. The exact SDM‐*Z* values were extracted from the local maxima (schematically indicated by black circles on the template brain) of the meta‐analytic difference map (Table 5) and for posterior ITG and dorsal IPL of the separate map for DO (Table 2; note that the dorsal IPL region was the submaximum of the left IPL cluster showing the highest summed SDM‐*Z* value across both orthographies).

### Left Occipitotemporal Cortex

The present meta‐analytic findings speak for a universal effect in the left OTC. Specifically, we identified convergent dyslexic underactivation for both deep and SO in the left posterior ITG (Fig. 2). Anatomically, overlapping underactivation was located in large portions of the left OTC and included the VWFA [Cohen et al., 2000, 2003; McCandliss and Noble, 2003]. At the classic location of the VWFA (*x* = −45, *y* = −57, *z* = −12, MNI), significant underactivation was evident in DO (SDM‐*Z* = 2.3) and SO (SDM‐*Z* = 2.3). Originally, dyslexic underactivation in the VWFA was suggested to reflect a deficit in fast parallel processing of known letter strings [Cohen et al., 2002]. Other interpretations assume a general impairment to link sensory information to higher level representations, such as phonology and semantics [Devlin et al., 2006; Price and Devlin, 2003], or a deficit in the build‐up of an orthographic word lexicon required for efficient memory‐based whole‐word recognition [Kronbichler et al., 2004].

The results of this meta‐analysis are largely in line with the only published cross‐linguistic functional neuroimaging study that directly compared dyslexic brain activation in three alphabetic languages with varying orthographic depth [Paulesu et al., 2001]. In this study, English, French, and Italian dyslexics exhibited common underactivation relative to nonimpaired readers in superior, middle, and inferior temporal, and middle occipital gyri (MOG). This meta‐analysis, however, localized common underactivation in DO and SO in the more ventral parts including left MTG, ITG, and MOG, but not in the left STG.

The identified universal effect is of interest with respect to the developmental aspect of neurocognitive models of dyslexia [e.g., Démonet et al., 2004; McCandliss and Noble, 2003; Pugh et al., 2001; Sandak et al., 2004]. A deficit in the left ventral OTC, presumably involved in visual‐orthographic whole‐word recognition, is assumed to result from a primary phonological deficit in the left dorsal TPC, presumably involved in serial grapheme–phoneme conversion. With respect to this developmental assumption, one might speculate that the present finding of orthography‐independent underactivation in the VWFA might be confounded by the adult studies. To account for this, we restricted the meta‐analyses to child studies (age means: 8–15 years). The additional analyses nevertheless identified dyslexic underactivation in OTC including the VWFA for DO and for SO. In a similar fashion, a recent age‐related meta‐analysis on dyslexic brain activation abnormalities identified underactivation in the left OTC already in dyslexic children [Richlan et al., 2011]. This finding supports the notion of an early OTC dysfunction in dyslexic readers, regardless of orthography.

A universal dysfunction of the left ventral OTC, including the VWFA in dyslexic readers is compatible with a newer model by Richlan [2012]. Based on meta‐analyses on brain dysfunction in children and adults across different orthographies [Richlan et al., 2009, 2011], this model assumes a primary dysfunction in the left ventral OTC. The left ventral OTC is proposed to be engaged in both lexical whole‐word recognition and serial sublexical grapheme–phoneme conversion [e.g., Richlan et al., 2010; Schurz et al., 2010]. The present meta‐analytic result of a universal effect in the left ventral OTC, together with evidence speaking for a key role of this area early on in reading development [e.g., Brem et al., 2010; Church et al., 2008; Gaillard et al., 2003; for a meta‐analysis, see Martin et al., 2015] and for a corresponding dysfunction already in young dyslexic children [e.g., van der Mark et al., 2009; Maurer et al., 2007; Olulade et al., 2015; Shaywitz et al., 2002; for a meta‐analysis, see Richlan et al., 2011] provide support for this new model.

The present finding of dyslexic underactivation in the VWFA independent of orthographic depth is also of interest with respect to a recent fMRI study on brain activation changes after specific training programs for different cognitive subtypes of dyslexia [Heim et al., 2015]. Heim et al. [2015] conducted four‐week trainings of phonology, attention, or visual word recognition according to the individual cognitive deficit profile of German dyslexic children. Besides some differences between the training programs, the VWFA was the only brain region where all three training programs led to substantial increase in reading‐related activation. This finding was interpreted as supporting the notion of the left ventral OTC as an integrative hub for different sources of information [Price and Devlin, 2011].

In addition to meta‐analytic findings speaking for a universal dysfunction in dyslexic readers in the VWFA, the extent of the underactivation clusters varied, depending on orthographic depth. Surprisingly, the most anterior segment (*y* = −42) of the visual word form system—as recently defined by anatomical connectivity [Bouhali et al., 2014]—was identified with significantly higher convergence of underactivation in SO compared with DO (Fig. 2, FFG). The maximum of this specific underactivation in left FFG was closer (Euclidean distance 6 mm) to a region identified with a length (i.e., number of letters) by lexicality (word vs nonword) interaction in German nonimpaired readers [Schurz et al., 2010]. Nonimpaired readers demonstrated increasing activation with increasing number of letters for nonwords but not for words. In contrast, German dyslexic readers generally showed lower activation in the left OTC and failed to show a modulation of activation in response to longer nonwords [Richlan et al., 2010]. In line with other studies pointing to a double function of the visual word form system [e.g., Braun et al., 2015b; Brem et al., 2010; Kronbichler et al., 2007, 2009; Ludersdorfer et al., 2013, 2016; Schurz et al., 2014a; Schuster et al., 2015], this pattern of activation was suggested to reflect an engagement in both lexical and sublexical processings.

One might speculate whether the present finding is confounded by the number of studies that used nonword stimuli. This is, however, unlikely as only two out of the nine SO studies that contributed to the left OTC cluster entered the meta‐analysis with a nonword contrast (Tables I and IV). In addition, the total number of studies using nonword stimuli was balanced across the two sets of studies. According to the double function of the visual word form system, the difference in the left FFG might reflect stronger reliance on serial decoding via grapheme–phoneme conversion in typical readers of SO, and thus, more consistent underactivation in dyslexic readers of SO.

In sum, the present meta‐analytic finding speaks for a universal effect of developmental dyslexia by indicating that dyslexic readers fail to adequately activate the left OTC system independent of the orthographic depth of their language. This universal effect is accompanied by specific left FFG underactivation in SO.

### Left Temporoparietal Cortex

With respect to the left TPC, the present meta‐analytic findings at first sight indicate a universal effect. Anatomically, common underactivation was evident in left dorsal IPL regions (Fig. 2), whereas more ventral left superior temporal/supramarginal regions exhibited underactivation exclusively in SO. This is interesting, as most neurocognitive models of dyslexia [e.g., Démonet et al., 2004; McCandliss and Noble, 2003; Pugh et al., 2001; Sandak et al., 2004] assume an extended left perisylvian TPC region with dyslexic underactivation centered in the left posterior STG/SMG. While the latter region is thought to be involved in phonology‐based reading processes [e.g., Jobard et al., 2003], the IPL was suggested to be part of the frontoparietal attention network interacting via top–down connections with language regions during reading [Shaywitz and Shaywitz, 2008].

The exact anatomical location of the overlap of convergent dyslexic underactivation in DO and SO was restricted to a dorsal segment of the left IPL. On closer consideration of this apparently shared left IPL cluster, most (six out of eight) DO studies reported dyslexic underactivation resulting from stronger task‐negative activation (i.e., deactivation relative to baseline) compared with nonimpaired readers. IPL deactivation was associated with active suppression of the default mode network [e.g., Laird et al., 2009] during reading. A similar finding of increased deactivation in the IPL was identified for dyslexic children compared with dyslexic adults and interpreted as reflecting increased cognitive effort during reading and reading‐related tasks [Richlan et al., 2011]. The dyslexic underactivation for DO in the bilateral IPL disappeared when all foci resulting from deactivation relative to baseline were excluded from the meta‐analysis. Consequently, the overlap was no longer present and the orthography‐specific bilateral intraparietal sulci (IPS) underactivation in DO disappeared from the difference map.

One may speculate that the increased deactivation in the IPL of dyslexic readers is the result of a particularly challenging in‐scanner task. Actually, four out of the six DO studies that showed increased task‐negative activation relative to baseline in the IPL of dyslexic readers used a word rhyme judgment task. Out of the two remaining studies that showed reduced task‐positive activation relative to baseline in the IPL of dyslexic readers, however, also one used this very same task [Cao et al., 2006]. In this study, dyslexic readers exhibited great difficulty as indicated by low accuracy both in absolute terms (45% correct) and relative to nonimpaired readers (79% correct). On average, the DO studies in which dyslexic IPL underactivation was due to increased task‐negative activation and the DO studies in which dyslexic IPL underactivation was due to reduced task‐positive activation differed neither with respect to absolute accuracy in dyslexic readers nor with respect to relative accuracy compared with nonimpaired readers. Thus, task type or task difficulty alone cannot fully account for the deactivation pattern in the IPL of dyslexic readers of DO.

The left TPC cluster identified with higher convergence of underactivation (resulting from reduced dyslexic task‐positive activation) in SO compared with DO was centered in a ventral part of the left SMG. This cluster was even more extended when all deactivation foci were excluded from the analysis. The left SMG has a well‐documented role in phonological processing [e.g., Braun et al., 2015a; Jobard et al., 2003; Sliwinska et al., 2012; Taylor et al., 2013; Vigneau et al., 2006]. During reading, engagement of the left SMG is thought to reflect serial mapping of orthography to phonology [Price, 2012]. Assuming that typical readers of SO rely more on rule‐based grapheme–phoneme conversion than typical readers of DO, the finding of higher convergence of underactivation in the left SMG in dyslexic readers of SO is not surprising. As evident from Table 1, the number of tasks that explicitly required phonological processing (e.g., rhyme judgment, phonological lexical decision, nonword reading) was equally high in both sets of studies with nine (DO) and eight (SO) out of 14 studies, respectively.

Overactivation in the left SMG (related to rule‐based grapheme–phoneme conversion) would be a feasible strategy in dyslexic readers of SO to compensate for underactivation in the left OTC (reflecting an orthographic lexicon deficit). Such overactivation, however, was not found in our meta‐analysis. Rather, dyslexic readers of SO showed overactivation in the left PRG, bilateral inferior frontal and caudate regions, left cingulate gyrus, and right cerebellum.

Besides the difference identified in the left TPC, we found the reverse pattern, that is, higher convergence of underactivation for DO compared with SO in the bilateral IPS. This difference, however, disappeared after exclusion of deactivation foci. The complex (de)activation pattern in the parietal cortex is of potential interest and calls for more fine‐grained anatomical characterization of parietal abnormalities in the original studies. Recent studies on receptor architectonics [Caspers et al., 2013], structural connectivity [Mars et al., 2011, 2012], and functional connectivity [Bzdok et al., 2013; Yeo et al., 2011] provided a detailed parcellation of the parietal cortex into various subdivisions and may be used to guide future fMRI analyses.

In sum, dyslexic underactivation seemingly common to DO and SO in the left dorsal IPL was actually based on increased deactivation in DO and reduced activation in SO. This pattern probably reflects different cognitive dysfunctions or compensatory mechanisms in the two sets of orthographies. The additional orthography‐specific underactivation in the left TPC around the ventral SMG presumably reflects increased reliance on serial sublexical decoding in typical readers of SO, and aberrant decoding processes in dyslexic readers of SO.

### Left Inferior Frontal Gyrus

With respect to the left IFG, the present meta‐analytic findings speak for orthography‐specific underactivation at different anatomical locations. Specifically, we found higher convergence of underactivation for DO compared with SO in the triangular part of the left IFG. The opposite pattern, that is, higher convergence of underactivation for SO compared with DO was identified in the orbital part of the left IFG and in the frontal operculum. The finding in the left pars orbitalis has to be interpreted carefully. On closer examination, the identified difference resulted from two contrary effects in DO and SO (Fig. 2), that is, overactivation in DO and underactivation in SO both below the threshold.

While the orbital part of the left IFG is associated with semantic retrieval [Binder et al., 2009; Bokde et al., 2001], the frontal operculum is associated with phonological processing [Fiez et al., 2006]. The triangular part of the left IFG was associated with both semantic and phonological processing [e.g., Vigneau et al., 2006]. In general, left inferior frontal regions were linked to a wide variety of linguistic processes including grapheme–phoneme conversion [e.g., Jobard et al., 2003], lexical access [e.g., Heim et al., 2013], phonological output computation [Taylor et al., 2013], speech planning [Price, 2012], and semantics [Binder and Desai, 2011]. Moreover, the left IFG is involved in not specifically linguistic processes like executive functions, working memory, reasoning, decision‐making, inhibition, attention, and emotion [Laird et al., 2011; Price, 2012; Richlan et al., 2014].

An interesting finding was the absence of common dyslexic abnormalities in left frontal regions. This could have been expected in particular with regard to our recent meta‐analysis on brain activation in typical readers of DO (English) and SO (Dutch, Finnish, German, Italian, Swedish). This meta‐analysis identified common reading‐related activation in a large cluster including left IFG and PRG regions [Martin et al., under review].

The orthography‐specific finding in the left frontal operculum is in line with our expectations. Specifically, we predicted underactivation in frontal regions predominantly in dyslexic readers of SO due to the reliance on a phonology‐based assembled reading strategy in typical readers (sublexical decoding via serial grapheme–phoneme conversion). This expectation was based in particular on a recent functional neuroanatomical model of developmental dyslexia [Richlan, 2012] proposing that underactivation in the left IFG reflects problems in accessing phonological output representations [Boets et al., 2013; Ramus, 2014; Ramus and Szenkovits, 2008]. This stands in contrast to previous English‐based models [e.g., Pugh et al., 2001] that propose dyslexic readers to exhibit overactivation in anterior regions to compensate for the deficits in the two posterior reading circuits (TPC and OTC).

The orthography‐specific finding in the triangular part of the left IFG (i.e., higher convergence of underactivation in DO compared with SO) might reflect another aspect of aberrant phonological processing in dyslexic readers of DO. In addition, the pars triangularis was shown to exhibit increased activation in response to exception words due to a selective increase in effective connectivity from the anterior OTC [Mechelli et al., 2005]. Exception words cannot be successfully read via sublexical phonological decoding, but have to be processed via an orthographic lexicon. Likewise, many other findings confirmed the role of the triangular part of the left IFG in phonological and semantic processing [e.g., Devlin et al., 2003; Poldrack et al., 1999; Price, 2012]. One might speculate that the functional abnormalities in frontal regions involved in both phonological and lexicosemantic reading processes may reflect the more severe dyslexic reading problems in DO (speed and accuracy deficit) compared to SO (speed deficit only).

### Left Precentral Gyrus

The left PRG was identified with convergent reading‐related overactivation in dyslexic readers in SO but not in DO, resulting in higher convergence in the direct statistical comparison. It is important to note that only 22 foci of overactivation were reported in the DO studies, whereas 86 foci of overactivation were reported in the SO studies. This imbalance is particularly problematic for the direct statistical comparison. Nevertheless, the inclusion of foci of overactivation increases the accuracy of the individual study‐specific maps and allows a complete assessment of universal and orthography‐specific dyslexic brain abnormalities.

The orthography‐specific effect in the left PRG was a surprising finding. Recent meta‐analyses on dyslexic functional brain abnormalities consistently identified the left PRG with overactivation in dyslexic children and adults [Richlan et al., 2009, 2011]. Specifically, overactivation near the mouth area [Fox et al., 2001] was thought to be independent of orthographic depth and to reflect compensatory reliance on covert articulatory processes during dyslexic reading from an early age on [Richlan, 2014]. The present finding indicates that the previously reported left PRG overactivation in dyslexic readers might have been largely driven by studies with German readers.

Additional support for an engagement of the left PRG specifically in SO comes from our recent meta‐analysis on brain activation in typical readers in DO (English) and SO (Dutch, Finnish, German, Italian, Swedish) [Martin et al., under review]. In this meta‐analysis, a similar left dorsal PRG cluster was exclusively identified with reading‐related activation in SO readers.

The left PRG was previously identified to be part of an articulatory network during language processing [Hickok and Poeppel, 2007]. During reading, it was identified with higher activation in response to nonwords compared with words [Price, 2012; Taylor et al., 2013]. In addition, this region showed a length effect (i.e., increasing activation with increasing number of letters) for nonwords in German nonimpaired [Schurz et al., 2010] and dyslexic readers [Richlan et al., 2010], speaking for an engagement of the left PRG in sublexical phonological decoding.

Due to the more consistent and regular nature of their orthography, dyslexic readers in SO can maybe rely more on compensatory articulatory mechanisms supported by the left PRG compared with dyslexic readers in DO. Consequently, they might rely less on alternative compensatory strategies (i.e., whole‐word guessing, semantic compensatory strategies) leading to a more consistent overactivation profile of the left PRG across tasks, studies, and individuals—reflected in the higher number of foci of overactivation in the original studies.

### Right Superior Temporal Sulcus

The right superior temporal sulcus (STS) was identified with higher convergence of underactivation in DO compared with SO. Notably, the separate meta‐analyses of DO and SO did not show convergent dyslexic functional abnormalities in this region. After lowering the voxel‐level statistical threshold to *p* < 0.05, however, the right STS was identified with convergent underactivation in DO and convergent overactivation in SO. Due to the contrary effects, the direct comparison (i.e., the meta‐analytic difference map) resulted in a statistically significant cluster in the right STS.

The right STS finding is of interest with respect to a meta‐analysis on structural brain abnormalities in dyslexia [Richlan et al., 2013]. The main finding across nine voxel‐based morphometry studies was consistent gray matter (GM) reduction in dyslexic readers in the right STG and left STS. The peak of right STG GM reduction was located near (Euclidean distance 15 mm) the right STS peak identified with orthography‐specific underactivation in the present meta‐analysis.

The right STS is thought to be an important region of the phonological network involved in the representation and processing of phonological information [Hickok and Poeppel, 2007]. It is activated during speech perception, speech production, and active maintenance of phonemic information. Furthermore, the right STS was shown to play a central role in the integration of auditory and visual information [van Atteveldt et al., 2004]. Dyslexic children were found to exhibit underactivation in this region in response to demands on letter‐speech sound integration [Blau et al., 2010]. This was interpreted as resulting from a failure to develop neural systems specialized for efficient interactive processing of auditory and visual linguistic inputs.

One might speculate that the higher convergence of right STS underactivation in DO compared with SO may be related to the severe phonological decoding deficit in dyslexic readers of DO. While dyslexic readers of DO suffer from a marked (non)word reading accuracy problem, dyslexic readers of SO usually exhibit slow but accurate (non)word reading. Interestingly, dyslexic readers of SO showed a tendency for overactivation in the right STS. Right hemisphere overactivation was previously interpreted as reflecting a compensatory mechanism for homologous left hemisphere underactivation [Pugh et al., 2000].

### Left Anterior Insula

The left anterior insula was identified with convergent overactivation in dyslexic readers of DO, but not in dyslexic readers of SO, resulting in higher convergence in the direct statistical comparison. This orthography‐specific finding has to be interpreted with caution, as consistency across studies was limited, with only two DO studies contributing to this cluster. Overactivation in the left anterior insula in DO stands in contrast to underactivation in the nearby frontal operculum in SO. Our orthography‐related meta‐analysis on brain activation in typical readers identified the left anterior insula with consistent reading‐related activation in both DO and SO [Martin et al., under review].

Left insula overactivation was previously interpreted as reflecting increased cognitive effort during dyslexic reading [Richlan et al., 2009]. Strikingly, one of the two DO studies contributing to the left anterior insula overactivation cluster reported extraordinarily low performance in dyslexic readers in a nonword reading task [Rumsey et al., 1997]. As evident from the Supporting Information, in this PET study, dyslexic readers exhibited significantly lower accuracy (39% correct) and significantly higher reaction times (2657 ms) compared with nonimpaired readers (82% correct and 1039 ms). In addition, variance in these measures was markedly higher in the dyslexic compared with the nonimpaired sample. These findings on task difficulty are in line with the notion of a role of the anterior insula in performance monitoring and conscious perception of errors [Ullsperger et al., 2010]. As part of the salience network, the anterior insula may be involved in the allocation of cognitive effort to important tasks. The dyslexic overactivation may reflect such effort during difficult reading tasks.

A surprising finding was that the insula was the only region with convergent overactivation in DO. In general, only relatively few foci of overactivation were reported in the included studies of DO (22 compared with 86 foci of overactivation in SO) and consistency across studies was limited. This might indicate more interindividual variability and less consistent compensatory strategies in dyslexic readers of DO compared with SO.

### Limitations

In general, meta‐analyses are limited by the characteristics of the included studies. In the case of orthography‐related meta‐analytic comparisons of dyslexic brain abnormalities, specific limitations exist regarding stimulus characteristics, in‐scanner activation tasks, behavioral performance (differences between studies and between groups), educational systems, characteristics of participants, criteria for the identification of dyslexic participants, and subtypes of dyslexia.

To increase comparability, we balanced the DO and SO studies with respect to the demands of the in‐scanner activation tasks. As evident from Table 1, the number of tasks that explicitly required phonological processing (e.g., rhyme judgment, phonological lexical decision, nonword reading) was equally high in both sets of studies with nine (DO) and eight (SO) out of 14 studies, respectively. Still, matching across studies in a meta‐analytical comparison is never perfect due to the limited number of available and suitable studies.

A similar issue concerns the difficulty of the in‐scanner activation tasks. As evident from Supporting Information, the mean accuracy was similar for DO and SO studies, *t*(23) = 0.994, *p* = 0.330, with 79.1% and 85.6% correct responses, respectively. It is reasonable to assume that the majority of the dyslexic participants were able to manage the in‐scanner activation tasks and that there are no systematic differences between the studies in DO and SO. Nevertheless, one should keep in mind the issues related to task type and performance when interpreting meta‐analytic differences between two sets of studies. Especially findings in regions commonly associated with error‐related activation (e.g., anterior insula, anterior cingulate cortex) have to be interpreted with caution.

With respect to diagnosis of dyslexia in the included studies of DO, some participants received a clinical diagnosis according to DSM‐IV [American Psychiatric Association, 2000] or ICD‐10 [World Health Organization, 1992], whereas others were classified as dyslexic when they had a documented history of reading difficulty in school or their reading performance (based on accuracy and/or speed) was about a standard deviation below the norm of a standardized reading test (see Supporting Information). In contrast, the majority of included studies of SO categorized dyslexics based on a marked reading speed impairment (below 10th percentile), which is considered the core criterion for diagnosing German dyslexic readers [Wimmer et al., 2000]. Thus, the diagnosis is based on poor reading accuracy and speed in DO and on slow reading fluency only in SO.

As aforementioned, the difference in the main criteria for the identification of dyslexic readers across DO and SO has to be taken into account in the interpretation of diverging brain activation findings. This difference, however, is a result of the different behavioral manifestations across orthographies and thus constitutes an inherent constraint. In contrast, the shared behavioral manifestation (and diagnosis criterion), that is, reading speed, is likely to be reflected in a shared brain activation abnormality. As evident from the results of our meta‐analysis, the shared reading speed deficit is most probably associated with underactivation in the left OTC including the VWFA.

An issue related to diagnosis concerns the presence of different subtypes or identification of specific cognitive profiles of dyslexic readers. It may be the case that the phonological subtype has a higher impact in DO, whereas the visual‐attentional subtype is more prevalent in SO. The identification of subtypes or cognitive profiles, however, is a relatively new development in the field and is hardly taken into account in functional neuroimaging studies of dyslexia. As evident from Supporting Information, none of the 28 studies included in the present meta‐analysis reported subtypes. Positive exceptions are the recent studies by Heim et al. [2015] and van Ermingen‐Marbach et al. [2013]. These studies showed both differences and commonalities across different cognitive subtypes of dyslexia. Whereas phonological and nonphonological dyslexics exhibited different levels of activation in left frontal and inferior parietal regions in a phonological lexical decision task [van Ermingen‐Marbach et al., 2013], three different types of training (specifically targeted at phonology, attention, or visual word recognition) in three corresponding subtypes resulted not only in an increase in reading performance in all groups but also in an increase in activation in a specific brain region, that is, the VWFA [Heim et al., 2015]. This finding is in line with the universal reading‐related dyslexic underactivation in the left OTC identified in the present meta‐analysis and its interpretation as reflecting the shared reading speed deficit in DO and SO.

It would have been of interest to test orthography‐specific developmental predictions derived from functional neuroanatomical models as proposed by Richlan [2014]. Currently, however, there are not enough functional neuroimaging studies conducted in different alphabetic languages to account for the precise and detailed age‐related (children vs adults) and task‐specific (phonological vs orthographic) aspects of these predictions. A single cross‐linguistic study with well‐matched samples, selection criteria, tasks, stimuli (words and nonwords), and an appropriate design would therefore be the method of choice.

Another interesting question refers to how the functional neuroanatomical manifestation of dyslexia is influenced by the properties of different writing systems (alphabetic, syllabic, and logographic). This was, however, beyond the scope of the present meta‐analysis. Although [e.g., Bolger et al., 2005] provided a seminal meta‐analysis targeting this issue, a new and updated meta‐analysis using state‐of‐the‐art methodology would probably provide novel insights into cross‐cultural effects of dyslexia.

## CONCLUSION

The present coordinate‐based meta‐analysis provides an objective quantification of commonalities and differences of dyslexic functional brain abnormalities (relative to nonimpaired readers) between alphabetic languages varying in orthographic depth. Dysfunctions and compensatory mechanisms of dyslexic readers in DO (English) and SO (Dutch, German, Italian, Swedish) were reflected in the degree, spatial extent, and exact anatomical location of under‐ and overactivation clusters.

Common underactivation in DO and SO was identified in the left OTC, including the VWFA. The universal OTC dysfunction, presumably reflecting a common phonological speed deficit, supports the idea that this area subserves both lexical whole‐word recognition and serial sublexical decoding (grapheme–phoneme conversion).

The left parietal cortex showed a specific pattern of dysfunctions. Underactivation in the left dorsal IPL resulted from stronger dyslexic task‐negative activation (i.e., deactivation relative to baseline) in studies of DO and from lower dyslexic task‐positive activation relative to baseline in studies of SO. This was interpreted as evidence for different underlying cognitive mechanisms.

The direct statistical comparison revealed orthography‐specific underactivation for DO in the left IFG pars triangularis, left precuneus, and right STS, together with exclusive overactivation in the left anterior insula. For SO, orthography‐specific underactivation was identified in the left FFG, TPC, IFG pars orbitalis, and frontal operculum, accompanied by exclusive overactivation in the left PRG.

In sum, the present meta‐analysis synthesizes and quantifies universal and orthography‐specific effects on dyslexic functional brain abnormalities during reading and reading‐related tasks in alphabetic writing systems. It broadens our understanding of the functional neuroanatomical signature of dyslexia and provides insights into compensatory mechanisms that may support remediation across languages varying in orthographic depth.

## Supporting information

Supporting Information
