# Supplementary material for: Dyslexic brain activation abnormalities in deep and shallow orthographies: A meta‐analysis of 28 functional neuroimaging studies
Source: Hum Brain Mapp. 2016 Apr 7;37(7):2676–99. doi: 10.1002/hbm.23202 (PMC5103175; doi:10.1002/hbm.23202)
Supplement: Supplementary file 1 — Supporting Information [file HBM-37-2676-s001.docx]

**SI**.

Supplementary information on the fMRI studies included in the meta-analysis.

|  |  |  |  |  | |  | |  |  |  |
| --- | --- | --- | --- | --- | --- | --- | --- | --- | --- | --- |
|  |  |  | In-scanner task performance  Dyslexic readers | | | In-scanner task performance  Non-impaired readers | | |  |  |
|  | Year | First author | Accuracy in % correct  mean (SD) | Reaction time in ms  mean (SD) | | Accuracy in % correct  mean (SD) | | Reaction time in ms  mean (SD) | Diagnostic instruments | Diagnostic criteria |
|  |  |  |  |  | |  | |  |  |  |
| *Deep orthographies* | | | |  | |  | |  |  |  |
|  | 2007 | Booth | 76.0 (14.0) | 1455 (304) | | 96.0 (4.0) | | 1276 (374) | TOWRE W reading efficiency (W reading speed), TOWRE NW decoding efficiency (NW reading speed), WJ-III W identification (W reading accuracy), WJ-III W attack (NW reading accuracy), WRAT spelling | mean of the four reading measures (accuracy & speed) < 95  (Standard scores *M* = 100, *SD* = 15) |
|  | 1999 | Brunswick | 90.1 (8.0) | - | | 96.6 (3.2) | | - | NART reading, WRAT reading & spelling, single W & NW reading, phonological measures | documented history of reading difficulty identified in childhood |
|  | 2006 | Cao | 44.8 (11.5) | 1593 (272) | | 79.5 (7.3) | | 1240 (351) | TOWRE W reading efficiency (W reading speed), TOWRE NW decoding efficiency (NW reading speed), WJ-III W identification (W reading accuracy), WJ-III W attack (NW reading accuracy) | mean on W & NW reading accuracy & speed < 95 |
|  | 2006 | Hoeft | 70.5 (17.2) | 2863 (411) | | 90.0 (10.5) | | 2445 (428) | WRMT W identification (W reading accuracy), WRMT W attack (NW reading accuracy) | W reading accuracy < 85 |
|  | 2007 | Hoeft | 86.1 (8.3) | - | | 97.3 (3.1) | | - | WJ spelling, WJ W identification (W reading accuracy), WJ W attack (NW reading accuracy), WRMT passage comprehension | W reading accuracy < 90, spelling or comprehension < 85 |
|  | 2010 | Hu | 86.5 (3.8) | 2369 (236) | | 88.6 (5.7) | | 1912 (263) | PAB phonological measures, WORD | diagnosis from prior educational assessment, reading, spelling, & phonology below the range of the control sample |
|  | | | | | | | | | | |
|  |  |  |  |  | |  | |  |  |  |
| **SI.** *Continued* | | | | | | | | | | |
|  |  |  |  |  | |  | |  |  |  |
|  | 2010 | Landi | 77.0 (17.0) | 1505 (246) | | 96.0 (6.0) | | 1332 (226) | WJ W attack (NW reading accuracy) | NW reading accuracy < 25th percentile or < 40th percentile with a prior diagnosis of reading disability |
|  | 2005 | McCrory | 99.4 (1.7) | 716 (111) | | 100.0 (0.0) | | 708 (88) | WRAT reading & spelling | history of reading difficulty in childhood or adolescence |
|  | 2008 | Meyler | 93.5 (12.3) | 4209 (969) | | 98.3 (3.8) | | 3159 (648) | TOWRE W reading efficiency (W reading speed), TOWRE NW decoding efficiency (NW reading speed) | W & NW reading speed < 30th percentile |
|  | 1996 | Paulesu | 95.0 (range 80-100) | - | | 98.0 (range 94-100) | | - | WRAT reading & spelling, NW reading & spelling, phonological measures | history of developmental dyslexia (dyslexia clinic) |
|  | 1997 | Rumsey | 39.0 (17.0) | 2657 (1115) | | 82.0 (7.0) | | 1039 (134) | GORT decoding accuracy and rate, WRAT reading & spelling | history of reading difficulty identified in childhood, DSM-IV criteria of developmental reading disorder |
|  | 2011a | Tanaka | 72.8 (19.1) | - | | 95.2 (6.7) | | - | WRMT W identification (W reading accuracy), WRMT W attack (NW reading accuracy), WRMT passage comprehension | W reading accuracy < 90 |
|  | 2011b | Tanaka | 79.6 (12.4) | - | | 94.9 (6.8) | | - | see above | see above |
|  | 2001 | Temple | 97.0 | 1000 | | 93.5 | | 850 | WRMT W identification (W reading accuracy), WRMT W attack (NW reading accuracy), WRMT passage comprehension | history of reading difficulty & W or NW reading accuracy < 85 |
|  |  |  |  |  | |  | |  |  |  |
| *Shallow orthographies* | | | |  | |  | |  |  |  |
|  | 2010 | Bach | 78.0 (8.4) | 2873 (246) | | 83.3 (11.1) | | 2787 (231) | SLT sentence reading speed, SRT spelling, BAKO | sentence reading speed < 25th percentile |
|  | 2006 | Brambati | - | 1540 (245) | | - | | 955 (95) | W, NW, & passage reading accuracy % speed, ADCL, phonological measures | W, NW, or passage reading (accuracy and/or speed) < 2 SD below the population or > 9 positive responses on the ADCL |
|  | 1999 | Georgiewa | 73.3 | 3550 | | 84.8 | | 1640 | ZLT reading, WRT spelling | reading < 1 SD & spelling < 2 SD below non-verbal intelligence |
|  | | | | | | | | | | |
| **SI.** *Continued* | | | | | | | | | | |
|  | | | | | | | | | | |
|  | 2004 | Grünling | 90.7 (7.7) | 2449 (562) | | 95.1 (5.0) | | 1732 (367) | ZLT reading, RT spelling, WRT spelling, phonological measures | group differences checked a posteriori |
|  | 2002 | Ingvar | - | | - | | - | - | reading comprehension, NW reading accuracy & speed | history of dyslexia in school |
|  | 2006 | Kronbichler | 95.6 (6.4) | 3329 (854) | | 98.1 (2.6) | | 2270 (938) | sentence, W, & NW reading accuracy & speed, spelling | sentence reading speed < 11th percentile |
|  | 2013 | Kronschnabel | 96.6 | | 530 | | 98.2 | 469 | SLRT II W & NW reading speed, SLS sentence reading speed, RT spelling | W or NW reading speed < 10th percentile in the past and < 20th percentile at present |
|  | 2011 | Maurer | 96.9 (9.2) | 713 (175) | | 99.5 (1.9) | | 605 (205) | SLRT & SLT W & NW reading speed, SLRT & DRT spelling | W & NW reading speed < 10th percentile |
|  | under review | Maurer | 90.4 (7.6) | 1366 (307) | | 92.3 (7.9) | | 1118 (259) | SLRT & DDD W & NW reading speed | W & NW reading speed < 10th percentile |
|  | 2011 | Pecini | 53.4 (19.5) | - | | 82.5 (17.9) | | - | BDE W & NW reading accuracy & speed, phonological measures | W & NW reading accuracy & speed < 1.5 SD below the population |
|  | 2010 | Richlan | 92.0 (7.7) | 1055 (302) | | 95.6 (6.3) | | 871 (262) | sentence reading accuracy & speed, SLRT W & NW reading accuracy & speed | sentence reading speed < 10th percentile |
|  | 2009 | Schulz | - | - | | - | | - | SLRT II W reading fluency | W reading speed < 10th percentile |
|  | 2009 | Van der Mark | 80.0 (9.0) | 1608 (252) | | 87.0 (9.0) | | 1196 (340) | SLRT W & NW reading speed, SLRT & DRT spelling | W reading speed < 10th percentile |
|  | 2010 | Wimmer | 95.1 (5.3) | 1269 (373) | | 96.0 (10.3) | | 973 (383) | sentence & text reading accuracy & speed, RT spelling | sentence reading speed < 10th percentile |
| *Note.* ADCL = Adult Dyslexia Check List, BAKO = Basiskompetenzen für Lese-Rechtschreibleistungen, BDE = Battery for the Diagnosis of Developmental Dyslexia and Dysgraphia, DDD = Dyslexia Differential Diagnostics, DRT = Diagnostischer Rechtschreibtest, GORT = Gray Oral Reading Test, NART = National Adult Reading Test, PAB = Phonological Assessment Battery, RT = Rechtschreibtest, SLRT = Salzburger Lese- und Rechtschreibtest, SLS = Salzburger Lese-Screening, SLT = Salzburger Lesetest, SRT = Salzburger Rechtschreibtest, TOWRE = Test Of Word Reading Efﬁciency, WJ-III = Woodcock Johnson III tests of achievement, WRAT = Wide Range Achievement Test, WORD = Wechsler Objective Reading Dimensions, WRMT = Woodcock Reading Mastery Tests, WRT = Westermann Rechtschreibtest, ZLT = Züricher Lesetest. | | | | | | | | | | |
